# Morphogenetic and Imaging Characteristics in Giant Cell Glioblastoma

**DOI:** 10.3390/curroncol29080422

**Published:** 2022-07-28

**Authors:** Cristian Ionut Orasanu, Mariana Aschie, Mariana Deacu, Liliana Mocanu, Raluca Ioana Voda, Theodor Sebastian Topliceanu, Georgeta Camelia Cozaru

**Affiliations:** 1 Clinical Service of Pathology, Department of Pathology, “Sf. Apostol Andrei” Emergency County Hospital, 900591 Constanta, Romania; mariana.aschie@365.univ-ovidius.ro (M.A.); mariana.deacu@365.univ-ovidius.ro (M.D.); lilianamcn@gmail.com (L.M.); raluca.voda@365.univ-ovidius.ro (R.I.V.); georgiana.cozaru@365.univ-ovidius.ro (G.C.C.); 2 Center for Research and Development of the Morphological and Genetic Studies of Malignant Pathology, “Ovidius” University of Constanta, 900591 Constanta, Romania; ts.topliceanu@365.univ-ovidius.ro; 3 Academy of Medical Sciences of Romania, 030167 Bucuresti, Romania; 4 Department of Pathology, Faculty of Medicine, “Ovidius” University of Constanta, 900470 Constanta, Romania; 5 Clinical Service of Pathology, Department of Genetics, “Sf. Apostol Andrei” Emergency County Hospital, 900591 Constanta, Romania

**Keywords:** CDKN2A, giant cell glioblastoma, imaging, immunohistochemistry, TP53

## Abstract

Giant cell glioblastoma is a rare tumor entity of IDH-wildtype glioblastoma. It is usually found in the pediatric population. We describe a particular case of a female patient diagnosed histopathologically with giant cell glioblastoma, who had two recurrences in different lobes of the same cerebral hemisphere, despite positive prognostic factors and appropriate treatment. We performed an immunohistochemical characterization of giant cell glioblastoma as well as an analysis of its aggressiveness using the cytogenetic markers TP53, CDKN2A, and TP73 using the FISH technique. The clinical picture was inconsistant, the suspicion being completely different initially. Paraclinical examination and imaging initially suggested a metastasis to the insular lobe. After surgery, histopathological and immunohistochemical examinations were the basis for the diagnosis. Despite the prognostic factors known so far in the literature, the aggressiveness denoted by multiple relapses and morphogenetic tests particularizes the case and improves the literature by bringing new information about this rare neoplasm of the central nervous system.

## 1. Introduction

According to the latest WHO classification, giant cell glioblastoma is a variant of glioblastoma IDH-wildtype along with gliosarcoma and epithelioid glioblastoma [1,2]. It accounts for up to 5% of all glioblastoma cases [3].

Little information is known about this tumor due to the rarity of cases described so far. It is more common in the pediatric population, with a frequency of approximately 3% of all neoplasms, as opposed to less than 1% in adults [3,4]. It develops at younger ages than glioblastoma, having a predominance over the male gender and a frequent localization in the temporal lobe [1,3]. It has a better prognosis than glioblastoma or gliosarcoma [5]. Microscopically, it is characterized by multiple bizarre multinucleated giant cells (sometimes over 20 nuclei) with atypical mitoses. It is also identified by a palisade appearance of ischemic necrosis and a reticulin network [1,2]. Treatment is based on maximal safe resection followed by adjuvant radiochemotherapy, which can increase the patient’s life expectancy [4].

Due to its rarity in the adult population, the rarity in the literature, and the lack of reports in our country, we decided to highlight and publish this case of double recurrence of a giant cell glioblastoma with some morphological and genetic peculiarities, but also a brief review of the anatomical–pathological and genetic diagnosis methods.

## 2. Case

### 2.1. Clinical Summary

A 58-year-old female patient was brought by relatives to the psychiatric ward for the following manifestations: insomnia, nausea, anxiety, vertigo, derealization, depersonalization, anomie, Broca’s aphasia, and olfactory illusions. Clinical and paraclinical investigations had suspected encephalitis. The patient was discharged with a slow favorable evolution.

Two months later, the patient presented with drowsiness, right hemiparesis, and headache. Computed tomography (CT) examination revealed a well-defined, isodense, iodophilic nodular lesion with a maximum diameter of 50 mm. It was located subcortial, on the left insular lobe associated with digitiform edema. The mass was suggestive for a secondary determination. She underwent surgery and the tumor fragments were examined histopathologically. The diagnosis was grade 4, giant cell glioblastoma. The patient received concomitant radiotherapy treatment (6 weeks with 2 Gy per session) and chemotherapy (temozolomide and carbamazepine 200 mg × 2/day). No tumor residue was identified on the control imaging. The clinical evolution of the patient was favorable during the treatment and for a short period after its completion.

**Figure 1:**
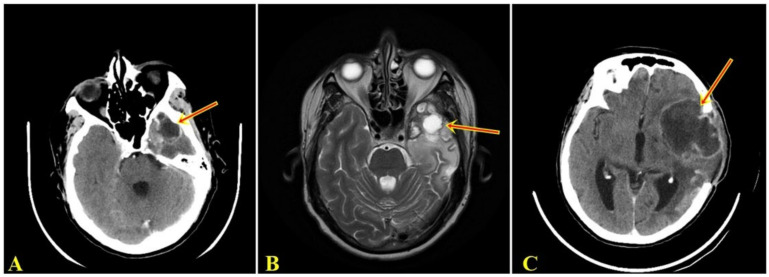
(**A**). Computed tomographic examination showing space-occupying mass, supratentorial, intranevraxial, with polylobular contour, with peripheral contrast, inhomogeneous structure, and a dense peripheral iodophilic component with axial diameters 42/34 mm, located left temporal that associates perilesional digitiform edema determining the asymmetry of the ventricular system. (**B**). MRI examination denotes a left temporal supratentorial tumor mass, with polylobate contour, in hypersignal T2 with inhomogeneous contrast, with axial diameters 49/44 mm, surrounded by edema. (**C**). Computer tomographic examination shows intraaxial lesion, with polylobate contour, with peripheral contrast, with inhomogeneous structure, with axial diameters of 75/57 mm, located left frontal with digitiform edema, with displacement of midline structures by 4 mm to the right, with slight proportion by postoperative bone breach.

Five months after the surgery, the patient presented to the neurosurgery department with dysphasia and left hemiparesis. CT and MRI examination revealed an intraneuraxial structure, on the left temporal lobe, with the appearance of a well-defined malignant tumor (Figure 1A,B). After the second surgery, the histopathological examination on the tumorectomy piece also pleaded for a giant cell glioblastoma.

Two months after the last surgery, the patient was readmitted to the neurosurgery department with fever and drowsiness. The CT examination showed a space-occupying mass, supratentorial, intranevraxial (left frontal lobe), with character of malignancy (Figure 1C). Before undergoing a new surgery, the patient died.

### 2.2. Pathology Findings

**Figure 2:**
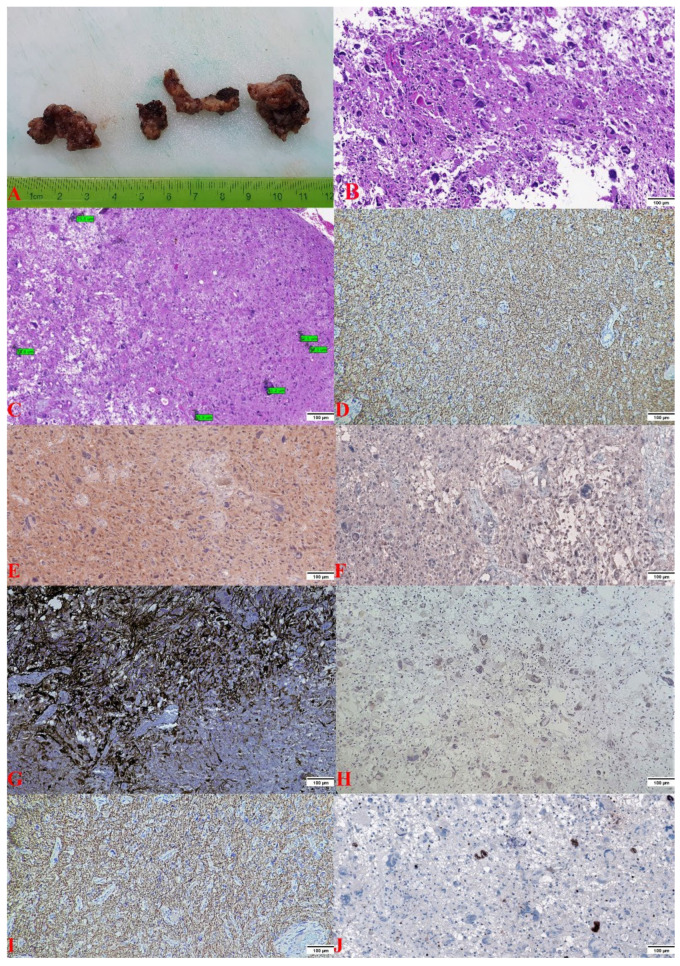
(**A**). The piece of cerebral tumorectomy of variable friable–elastic consistency, which in the section has small cavitary areas with the presence of hemorrhagic areas. (**B**). Dense cellular tumor proliferation with numerous atipical cells and multinucleated giant cells, palisade necrosis, endothelial vascular hyperplasia, and areas of hemorrhage (hematoxylin–eosin staining, ×100). (**C**). Morphometric analysis reveals multinucleated giant cells with dimensions between 50 and 81 µm. (**D**). GFAP stain: intense positive cytoplasmic (×100). (**E**). S100 stain: moderately positive cytoplasmic (×100). (**F**). Vimentin stain: moderately positive cytoplasmic (×100). (**G**). Nestin stain: intense positive cytoplasmic (×100). (**H**). ATRX stain: moderately positive nuclear (×100). (**I**). Synaptophysin stain: intense positive membrane (×100). (**J**). Ki-67 stain: intense positive in 20% of nuclei (×100).

**Figure 3:**
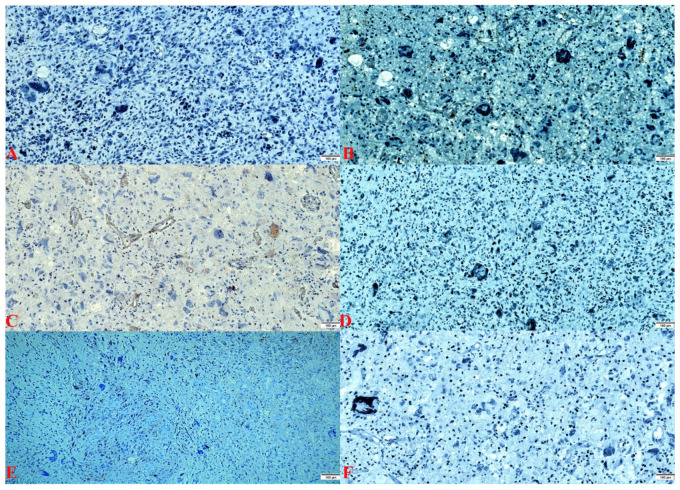
(**A**). IDH1 R132H: stain negative (×100). (**B**). SOX-10 stain: negative (×100). (**C**). EGFR stain: endothelial positive and negative in the tumor population (×100). (**D**). p53 stain: negative (×100). (**E**). EMA stain: negative (×100). (**F**). NeuN stain: negative (×100).

Macroscopically, a lesion with dimensions of 5/5/1.5 cm, of low consistency with elastic areas, and reddish-gray with yellow areas was observed (Figure 2A). During the sectioning, small cavity spaces occupied by saguinolent liquid were highlighted. Both histopathological evaluations highlighted a proliferation of glial cells with moderate cyto-nuclear pleomorphism to which numerous multinucleated giant cells were associated, without the presence of macrophages. In addition, glomeruloid-type microvascular proliferation was observed as well as limited areas of necrosis with perinecrotic pseudopalysis (Figure 2B). Morphometric analysis revealed multinucleated giant cells with dimensions between 50 and 81 µm (Figure 2C). Immunohistochemical tests showed immunopositivity for: GFAP (+++), S100 (++), Vimentin (++), Nestin (+++), ATRX (++), Synaptophysin (+++), and KI-67 (20%) (Figure 2D–J). Immunohistochemical tests showed immunonegativity for IDH1 R132H (–), SOX10 (–), EGFR (–), p53 (–), EMA (–), and NeuN (–) (Figure 3). Each immunohistochemical section was tested by a positive control slide.

**Figure 4:**
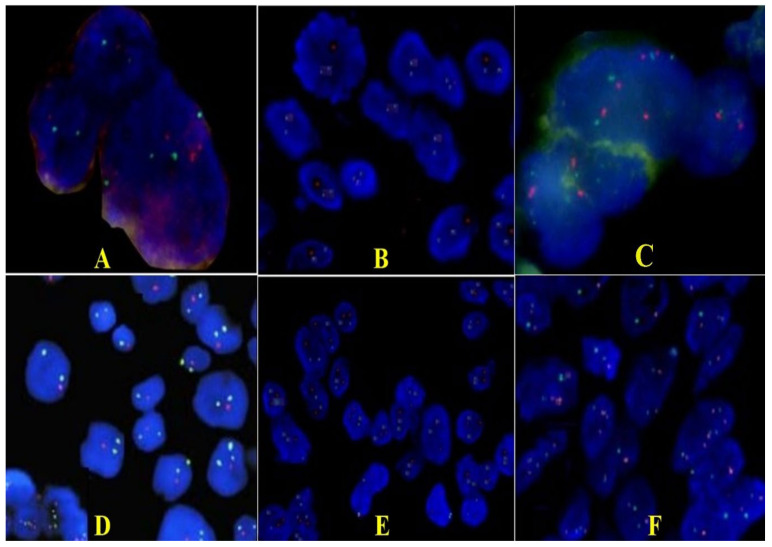
Representative FISH results for p53 (**A**,**B**), CDKN2A (**C**), p73, GLTSCR1, and GLTSCR2 (**D**–**F**) expression on giant cell glioblastoma (×100). (**A**,**B**). Photomicrographs of tumors showing hemizygous deletion of TP53/D17S655 locus: a single red signal in 78% of the tumor nuclei and paired green signals for D17Z1. (**C**). CDKN2A gene showing hemizygous deletion. (**D**). Only one red signal (deletions of the 1p36.32 region including the TP73) and two green signals (1q25.2 region) are detected in each nucleus. (**E**). Section of a tumor showing normal disomic complement of 19q13/19p13 regions in majority of the cells; some nuclei have less than two copies because of the truncation artifact encountered in thin tissue sections. (**F**). Loss of 19q13 (single red signals in nuclei and paired green signals for 19p13) that cover regions including the GLTSCR1 and GLTSCR2 genes.

Tissue samples were used to determine p53 (TP53), CDKN2A, p73, and glioma tumor suppressor candidate region genes 1 and 2 genes (GLTSCR1 and GLTSCR2) status by fluorescence in situ hybridization (FISH). We chose these genes because they are involved in apoptotic activity and have an impact on the aggressive potential. In the case presented, most cells (78% of analyzed cells) had one red and two green signals, which means a p53 gene hemizygous deletion status (Figure 4A,B). Only a few nuclei showed only two green signals, signifying a homozygous deletion of the p53 gene. The rest of the evaluated cells showed a disomic status. In the case of the CDKN2A gene, the cells had a green signal and two red ones, which means a status of hemizygous deletion of the gene (Figure 4C). After hybridization with the 1p36 probe (red signal) and the 1q25 probe (green signal) showed deletion of the 1p36 region. That means the presence of the p73 gene deletion was in approximately 85% of the nuclei; the rest had a disomic status (Figure 4D). Regarding the second sample, results showed the absence of deletion in the 19q13/19p13 regions in most of the investigated nuclei. In some areas, nuclei with hemizigotic deletion of the 19q13.33 region were noted (Figure 4E,F). However, we cannot say that this was a co-deletion of 1p and 19q, because we identified only deletions of some regions, not of the entire chromosomal arm, but we appreciated that this case associated only the deletion of the p73 gene.

## 3. Discussion

This tumor entity is more common in the pediatric population and approximately 100 cases have been identified so far [6]. Extremely rare are the cases found in the adult population, usually detected in people under 40 years of age, especially in males [3,6]. In our case, the presence of giant cell glioblastoma was in a woman in the sixth decade of life. A study conducted by Jin MC et al. indicates that the female gender is a positive prognostic factor [7].

The clinical presentation is unsuggestive and can be confused with the symptoms of an infectious or inflammatory disease, which happened in our case, the initial diagnosis being encephalitis [8]. The clinical picture may suggest an expansive intracranial process that may be eloquent from the onset of the disease or over time by the appearance of manifestations such as seizures or paresis (50% of cases) [6].

Imaging aspects of giant cell glioblastoma indicate a low potential for aggressiveness, being described as a circumscribed lesion that can be misdiagnosed as metastasis [1]. This fact was found imagistically in the first instance in our case. The same similar aspect, with benign, but deceptive imaging characteristics, has been identified in other tumors of the nervous system, a malignant ependymoblastoma that mimics a pilocytic astrocytoma [9]. Due to this well-defined aspect, the post-therapeutic survival rate is 17.55 months, a higher rate than a glioblastoma [7]. It is assumed that this is primarily due to the greater ability of the surgical therapeutic approach, which can leave free surgical margins [10]. In our case, the patient survived 9 months from the first manifestations and 7 months after the initial diagnosis and first trimodal treatment. This is also due to the particularity of the case of double recurrence in different ipsilateral brain lobes in a short period of time, a peculiarity not found in the literature.

The histopathological features of the presented case are the classic ones, but what is added to the literature is the immunohistochemistry corroborated with the genetic tests. The positive reactions to GFAP and S100, as well as the negative reaction to ATRX, presented in the study, are in line with the data in the literature [2]. The reaction to vimentin is more associated with the mesenchymal component of gliosarcoma, but can also be identified in giant cell glioblastoma, its role having implications in proliferation and invasiveness [2,5]. Despite the clinical history similar to secondary glioblastoma (mutant IDH), the wildtype status of IDH supports de novo development as well as the aggression [11].

Of note is the Ki-67 nuclear proliferation index. Multinuclear giant cells are not formed by fusion, they are cells blocked in mitosis, so they do not appear to have proliferative activity [12]. However, there are situations in which immunopositivity has been observed even in the bizarre nuclei of giant cells [13]. In the case of giant cells glioblastoma, the proliferation rate is 31–89%. The presence of an increased index in mononuclear cells confers an aggressive behavior of the tumor [12,13]. The proliferation rate in our case was 20%. This index suggests a less aggressive behavior. This aspect is in contradiction with the evolution of this case, marked by recurrences and rapid evolution towards exitus.

An initial stage in the genesis of giant cells glioblastoma is the mutation of the TP53 gene. This aspect is found in 75–89% of cases. The immunohistochemically nuclear reaction for p53 is found in over 70% of cases [12,14]. In our case, both immunohistochemical p53 and the FISH TP53 genes are negative, respectively, deleted. This feature gives the appearance of null type and can explain the aggressive and recurrent nature of the tumor.

According to an analysis by Oh JE. et al., they noted that the most important genetic events were the loss of chromosome 19q and mutations of TP53, very rarely were identified TERT mutations or EGFR amplifications. Similarly, our case showed p53 mutations, but the loss of chromosome 19 was not a predominant status in the nuclei [11].

Several studies have shown that concurrent losses (co-deletion) of the 1p36.32 and 19q13.33 regions are reported in approximately 80% of oligodendrogliomas and some glioblastomas [15]. Furthermore, 1p and 19q co-deletion has a strong prognostic factor in these diseases, where it is associated with improved prognosis and responsiveness to therapy [16]. Comparing to data from the literature, our case is notable for the absence of co-deletion 1p36.32 and 19q13.33 regions, but still shows the hemizygous deletion of the p73 gene.

The mutation of the CDKN2A gene is extremely rare (3%), and its presence associated with clinical evolution has led some researchers to consider these tumors as an intermediate between IDH wildtype and mutant IDH glioblastoma [14]. The mutant status gives a reserved prognosis to the patient, this was obvious in our case as well [17].

Two aspects that complete the image of a low survival are noted in the immunonegativity to SOX10 and the increased intensity of the Nestin marker. In gliomas, Sox-10 is recognized as a marker of oligodendroglial differentiation, which is associated with increased survival [18]. The loss of SOX-10 function leads to a significant decrease in survival, increasing tumor invasiveness and immune cell infiltration [19]. Nestin is a marker of neuroepithelial stem cells, whose activity decreases after cell differentiation. It has a strong correlation with the high histopathological degree of glial tumors, considered by some authors as an important marker of negative prognosis in terms of survival [20].

The immunonegativity of EMA and NeuN was indicated by the study of Shrestha S. et al., the last marker having as a role the differential diagnosis of a pleomorphic xantoastrocytoma [6]. Thus, the differential diagnosis should be made with pleomorphic xantoastrocytomas, which resemble the macroscopically well-defined appearance, the presence of giant cells, but differs by the presence of macrophages and immunopositivity to neuronal nuclear antigen, neurofilament protein, and synaptophysin [12].

The standard treatment is trimodal: complete surgical resection and concomitant chemoradiotherapy. Temozolamide 75 mg/m^2^ is administered daily, until the end of radiotherapy doses, not more than 49 days. A total dose of 60 Gy divided into 2 Gy is applied, five days out of seven, for 6 weeks. This should be followed by six cycles of adjuvant temozolamide treatment (5 days in a 28-day cycle) [21,22]. The same treatment was performed by our patient, along with a symptomatic treatment, but without success. The failure can be attributed to gene alterations that have given the tumor a very aggressive status. We can conclude that in our case, the genetic abnormalities have given a poor prognosis and a very aggressive status, which could not be counteracted even by an adequate treatment, recognized worldwide.

## 4. Conclusions

A non-suggestive clinical picture corroborated with an eloquent imaging for a tumor with a high degree of malignancy are major impediments in the diagnosis of a giant cell glioblastoma. It should be noted the importance of histopathological, immunohistochemical, and genetic tests in establishing a complete diagnosis and are implicit in providing effective treatment. Even in the presence of some positive prognostic factors and adequate treatment, its aggression is marked both by its ability to recur and by leading to exitus in a short period of time. The study outlined its objectives by presenting a rare case of giant cell glioblastoma as well as compiling a diagnostic panel that assesses aggression.

## Data Availability

The data presented in this study are available on request from the corresponding author.
